# Upper gastrointestinal cancers in Lynch syndrome: the time for surveillance is now

**DOI:** 10.18632/oncoscience.525

**Published:** 2021-03-21

**Authors:** Shria Kumar, Bryson W. Katona

**Affiliations:** ^1^Division of Gastroenterology and Hepatology, University of Pennsylvania Perelman School of Medicine, Philadelphia, PA, USA

**Keywords:** Lynch syndrome, upper gastrointestinal cancer, gastric cancer, surveillance

Lynch syndrome (LS) is among the most common hereditary cancer predisposition syndromes. Affected individuals carry a pathogenic or likely pathogenic germline variant in a DNA mismatch repair gene (*MLH1, MSH2, MSH6, PMS2* or *EPCAM*), predisposing them to high lifetime risks of malignancies [1]. Colorectal cancer is the most common gastrointestinal (GI) malignancy in LS, but there is also increased risk of gastric and small intestinal cancers. LS confers a cumulative lifetime risk of up to 9% for gastric cancer (GC) (compared to 0.9% in the general population) and up to 11% for small bowel cancer (SBC) (0.3% in the general population) [2]. Despite these high lifetime risks, there is a lack of consensus regarding surveillance of upper GI (UGI) cancers. While colorectal cancer surveillance guidelines are robust, UGI cancer surveillance has many areas of uncertainty, including: age of surveillance initiation, surveillance intervals, and whether all individuals with LS or only gene-specific subgroups should undergo surveillance [2-8]. The result is variable practice patterns and a potential source of uncertainty for patients and providers alike.

In our tertiary care referral center we offer UGI cancer surveillance to all individuals with LS starting at age 30 with mucosal biopsies of the gastric antrum and body. We recommend a 1-2 year surveillance interval, with upper endoscopy performed concurrently with a lower GI surveillance procedure. We recently described our outcomes from UGI surveillance in LS. [9] Among 295 with LS, 217 (73.6%) underwent at least 1 upper endoscopy, with 11 (3.7%) patients being diagnosed with an UGI cancer: one esophageal squamous cell carcinoma, six gastric adenocarcinomas, and four duodenal adenocarcinomas. Among the 11 persons who developed an UGI cancer, 5 of these cancers were detected during surveillance, while the remainder were discovered during work-up of symptoms.

Our findings highlight a number of important points. First, UGI cancers detected on surveillance were detected at an early stage. Compared to those who underwent endoscopy for symptomatic indications, surveillance detected cancers were more likely to be detected as a (stage I), 80% vs 33.3%. Identifying UGI cancers at early stages allows for potentially curative surgical therapy.

Second, our findings suggest that a short surveillance interval is warranted. Of the 5 individuals with UGI cancer detected on surveillance, 4 (80%) had a prior surveillance upper endoscopy within 2 years of their cancer diagnosis. While our findings are relatively limited in power, they support that a shorter UGI surveillance interval may be warranted, and also hint at possible accelerated carcinogenesis for LS-related UGI cancers. While colorectal cancers in LS follow an accelerated adenoma-to-adenocarcinoma pathway, it is unclear if a similar pathway applies to LS-related UGI cancers, though if this is confirmed, would support shorter surveillance intervals [1].

Third, factors previously associated with LS-related GC include male sex, *MLH1/MSH2* pathogenic variant, and family history of GC [10], however we found no significant differences between those with and without UGI cancers with respect to these factors. This suggests that at this time risk factors alone are not sufficient to determine which individuals should undergo UGI surveillance, and therefore all individuals with LS should be considered for surveillance.

Our findings were supported by a subsequently published study by the German Consortium for Familial Intestinal Cancer, evaluating the utility of upper endoscopy for GC detection in 1128 individuals with LS [11]. In this study, 47 (4.2%) individuals were diagnosed with GC, and similar to our findings, GC detected on surveillance were found at early stages (83% stage I) compared to cancers detected outside surveillance (25% stage I). The majority (68%) of individuals with detected GC did not have a family history of GC, and 11% were younger than age 40. Additionally, similar to our results, 83% of the patients with surveillance detected cancers had an upper endoscopy within the prior two years, again alluding to the possibility of accelerated UGI carcinogenesis in LS.

Despite the emergence of studies evaluating this topic, multiple areas of uncertainty persist. The age at which to start surveillance remains unclear. In our cohort UGI cancer in LS was diagnosed as young as 41 years and 11% of patients with UGI cancer in the German Consortium were under 40 years old, thus supporting that upper GI surveillance initiation should occur prior to age 40. Surveillance intervals also remain controversial. Recent data supports short surveillance intervals, with the majority of LS-related UGI cancers being detected within 2 years of a prior surveillance upper endoscopy [9, 11]. In the setting of a shorter surveillance interval, we believe that UGI surveillance procedures should always be performed concurrently with a lower GI surveillance procedure, to minimize procedural burden. However, the cost-effectiveness of this strategy remains uncertain. Finally, there are wide variations in reported lifetime risk for GC and SBC, dependent on the specific LS gene. For example, while *MSH1* confers a 11% risk of SBC by age 80, the risk for *PMS2* carriers is reported to be 0.1-0.3%, essentially that of the general population [2]. It is possible that UGI cancer risk estimates in LS will change as more affected families are identified, however in the meantime, questions remain on whether UGI surveillance should be tailored by pathogenic variant.

There will undoubtedly be future studies of UGI surveillance outcomes in LS, and as data accumulates, we hope for more clarity regarding its effectiveness. For now, given recent evidence in the field, we believe that UGI surveillance should be incorporated into the cancer risk management plan of individuals with LS.

**Figure 1:**
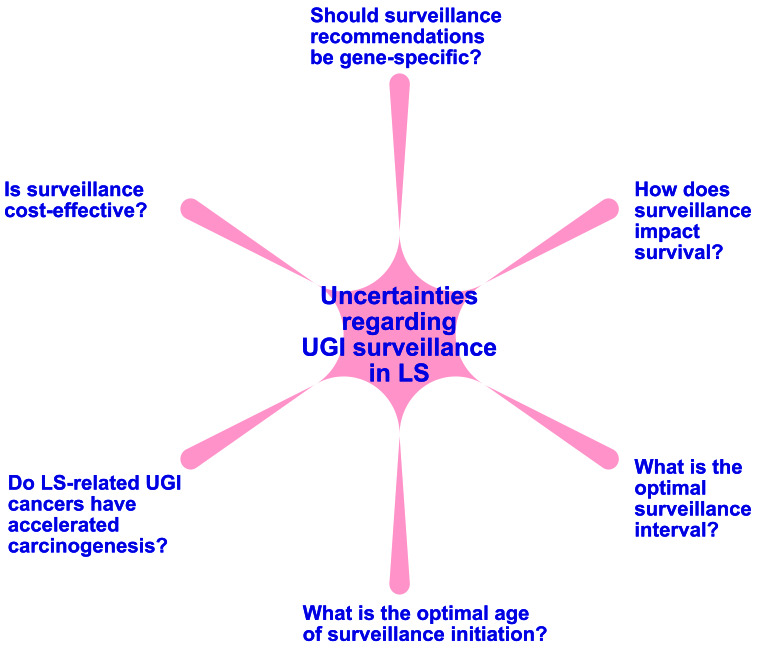
Uncertainties regarding surveillance for upper GI cancers in Lynch syndrome
